# Sex differences in neural and behavioral signatures of cooperation revealed by fNIRS hyperscanning

**DOI:** 10.1038/srep26492

**Published:** 2016-06-08

**Authors:** Joseph M. Baker, Ning Liu, Xu Cui, Pascal Vrticka, Manish Saggar, S. M. Hadi Hosseini, Allan L. Reiss

**Affiliations:** 1 Center for Interdisciplinary Brain Sciences Research, Division of Brain Sciences, Department of Psychiatry and Behavioral Sciences, School of Medicine, Stanford University, 401 Quarry Rd., Stanford CA, 94305 USA; 2 Max Planck Institute for Human Cognitive and Brain Sciences, Department of Social Neuroscience, Stephanstraße 1A, Leipzig, 04103 Germany; 3 Department of Radiology, School of Medicine, Stanford University, 1201 Welch Rd., Palo Alto, CA, 94304 USA

## Abstract

Researchers from multiple fields have sought to understand how sex moderates human social behavior. While over 50 years of research has revealed differences in cooperation behavior of males and females, the underlying neural correlates of these sex differences have not been explained. A missing and fundamental element of this puzzle is an understanding of how the sex composition of an interacting dyad influences the brain and behavior during cooperation. Using fNIRS-based hyperscanning in 111 same- and mixed-sex dyads, we identified significant behavioral and neural sex-related differences in association with a computer-based cooperation task. Dyads containing at least one male demonstrated significantly higher behavioral performance than female/female dyads. Individual males and females showed significant activation in the right frontopolar and right inferior prefrontal cortices, although this activation was greater in females compared to males. Female/female dyad’s exhibited significant inter-brain coherence within the right temporal cortex, while significant coherence in male/male dyads occurred in the right inferior prefrontal cortex. Significant coherence was not observed in mixed-sex dyads. Finally, for same-sex dyads only, task-related inter-brain coherence was positively correlated with cooperation task performance. Our results highlight multiple important and previously undetected influences of sex on concurrent neural and behavioral signatures of cooperation.

Research into the neuroscience of social behavior has highlighted a frontal-temporal network in the brain that underlies social cognition^1^,^2^,^3^,^4^. Converging evidence from this field indicates that multiple sub-regions of the prefrontal cortex (PFC) are largely concerned with determining future behavior on the basis of anticipated value of different social actions. Using fMRI, distinct cognitive functions related to social value judgments have been identified within the anterior rostral medial PFC^5^,^6^,^7^, posterior rostral PFC^8^,^9^,^10^, and orbital medial PFC^8^,^10^,^11^,^12^. Similar to the PFC, distinct sub-regions of the occipital-temporal-parietal cortex have been implicated in cognitive functioning related to person-centered social processing such as interpretation of biological motion^13^, perception of bodily action and goals^2^,^3^,^14^,^15^,^16^, theory of mind^2^,^3^,^14^,^15^,^16^, and computational processes associated with the sense of agency^15^,^17^,^18^,^19^,^20^. An important drawback to this research is that the majority of studies investigating the neural correlates of social cognition are conducted on a single person in isolation. As a result, very little is currently understood about how the human brain responds to real-world social interaction, and how such responses may relate to cooperation behavior^21^. Recent advancements in simultaneous neuroimaging of multiple brains (i.e., hyperscanning) using functional near-infrared spectroscopy (fNIRS) have revealed increases in inter-brain coherence that results from naturalistic cooperation between two people^22^,^23^,^24^. That is, when engaged in a computer-based cooperation task, the right prefrontal cortex within both members of an interacting dyad becomes synchronized during the task compared to rest^22^,^23^. Furthermore, increases in coherence have been associated with increased task performance^22^,^23^, suggesting that a mechanistic association may exist between inter-brain coherence and cooperative behavior.

Recent findings suggest that inter-brain coherence during cooperation may be mediated by the sexual makeup of the interacting pair. Indeed, researchers have hypothesized that disparate evolutionary pressures experienced between males and females may have resulted in sex-related differences in cooperation within modern humans^25^,^26^,^27^. That is, an evolutionary history rich with hunting and warfare may have resulted in male/male dyads tendency towards a cooperation strategy that differs from that of females, who historically maintained different social roles. Interestingly, Cheng and colleagues^23^ report mixed-sex dyads exhibited significant inter-brain coherence in regions of the prefrontal cortex during cooperation, whereas same-sex dyads did not. Furthermore, increased inter-brain coherence in mixed-sex dyads was associated with increased cooperation task performance. As the authors report, these findings suggest that “different neural processes underlie cooperation between mixed-sex and same-sex dyadic interactions”. These findings raise important empirical questions regarding the influence of sex on neural and behavioral signatures of cooperation and highlight the utility of fNIRS hyperscanning in addressing sex differences in social interactions.

A significant shortcoming of the study reported by Cheng and colleagues is the inability to identify the role of sex on signatures of inter-brain coherence within regions of the temporal cortex. Given our understanding of the different socio-cognitive processes expressed throughout the frontal-temporal network, it is likely that disparate patterns of inter-brain coherence may emerge throughout this network between male/male, female/female, and male/female dyads engaged in cooperation. The identification of disparate patterns of inter-brain coherence throughout the frontal-temporal social network between same- and mixed-sex dyads may provide important information regarding the underlying source of sex-related differences in cooperation. Here, we test the hypothesis that the sexual make-up of a cooperating dyad moderates signatures of cortical activation and inter-brain coherence within multiple fontal-temporal brain regions associated with social cognition. To address this hypothesis, we employed fNIRS hyperscanning to concurrently image regions of the right prefrontal and right temporal cortices as same- and mixed-sex dyads engaged in a computer-based cooperation task.

## Methods

### Participants

A total of 222 participants (N_female_ = 110) were recruited for participation. Each participant completed a computer-based cooperation task with either a male or female partner (male/male = 39, male/female = 34, female/female = 38). Each participant was unacquainted with his or her partner and participants were not permitted to interact prior to participation. Age and ethnicity were not matched across participants. All participants were right handed and had normal or corrected to normal vision and hearing. Informed consent was obtained from both dyad members prior to participation. The Stanford University Institutional Review Board approved all aspects of our experiment. The experiment was performed in accordance with all relevant guidelines and regulations.

### Cooperation Task

**Figure 1:**
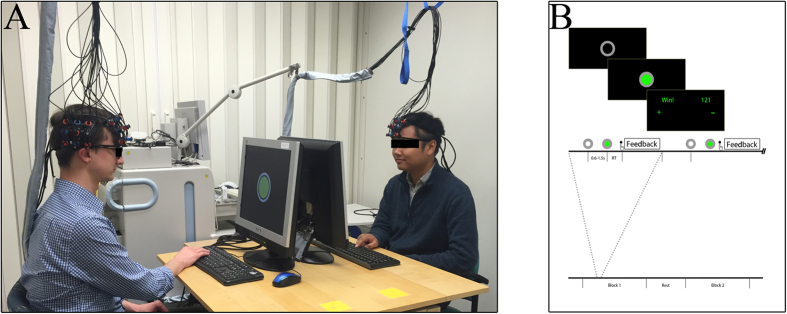
Participant arrangement and cooperation task structure. (**A**) Participants were sat on opposite sides of a square table, in front of individual monitors and keyboards. (**B**) Trial stimulus sequence. Both participants were seated in front of their own computer screen and keyboard. Each trial began with a hollow gray circle appearing in the center of the screen for an unpredictable duration between 0.6 and 1.5 seconds. An abrupt change in the color of the circle from black to green initiated the participant’s response. Following both responses, a “win” or “lose” message was presented, depending on whether the inter-response latency of both participants was less than 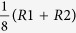, where R1 and R2 are the response times for both participants respectively. **A** ‘+’ and ‘−’ sign was also presented, which indicated the faster/slower responder for each trial. The symbol on both participants’ left hand side of the screen corresponded to their response speed relative the their partner. Two 40 trial cooperation task blocks were separated by a 30 second rest period. Task-related inter-brain coherence was calculated as the average coherence during both task blocks minus coherence during rest 
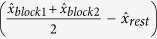
.

The participants were seated on opposite sides of a square table, in front of a separate computer screen and keyboard (Fig. 1A). Task instructions and three practice trials were provided prior to beginning. Each trial began with a hollow grey circle appearing in the center of their computer screen for an unpredictable duration between 0.6 and 1.5 seconds. A rapid change in the color of the circle from black to green initiated a button press response from both participants. In order to win points for each trial, the participants had to match their button presses closely in time so that the latency between button presses was less than 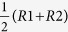, where R1 and R2 are the response times for both participants respectively. Immediately following the slower member’s button press (i.e., second button press of the trial) a ‘+’ and ‘−’ sign was presented in the top portion of both participant’s screens and which identified the faster/slower responder for each trial. The symbol on both participants’ left hand side of the screen corresponded to their response speed relative to their partner. Thus, participants could use this information to adjust their response speed on subsequent trials.

The cooperation (i.e., coordination) task consisted of two 120 s task blocks, separated by a 30 s rest period. A total of 40 trials were completed within each task block. Dependent on the inter-response latency within each trial, a “Win” or “Lose” label was provided at the top of both participants’ screens, along with their point total. Each dyad began the task with 100 points and was instructed to earn as many points as possible throughout the game. Each dyads average performance (i.e., #wins/40) was calculated for both task blocks (Fig. 1B).

### fNIRS Optode Arrangement

**Figure 2:**
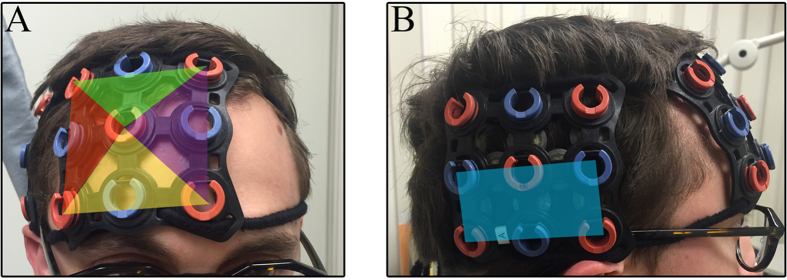
fNIRS regions of interest. (**A**) The medial optode column of a 3 × 3 fNIRS optode patch was placed over the midsagital line and directly above the brow. Four three-channel regions (colored triangles) were identified a priori. The anterior edge of a 2 × 3 fNIRS optode patch as placed over the interauricular arc and directly above the T4 10/20 location. (**B**) A single region of interest (blue rectangle) was established in the temporal region.

A continuous wave fNIRS (ETG-4000, Hitachi, Japan) was used to assess cortical hemodynamic activity in each dyad member’s right PFC and right temporal cortex. The ETG-4000 system provides users with versatile probe arrangements that may accommodate 24, 48, or 52 recording channels depending on the pre-defined probe configuration selected (i.e., 3 × 3, 3 × 5, 4 × 4, or 3 × 11). In previous studies^22^,^23^, a single 3 × 5 optode patch (i.e., 22 recording channels) was situated over the hyperscanning participants’ PFC. Here a 3 × 5 optode patch was broken down into one 3 × 3 (i.e., 12 channels) and one 2 × 3 (i.e., 7 channels) optode patch. For each dyad member, the medial edge of the 3 × 3 optode patch was aligned over the inion-to-nasion midsagital line, and the inferior edge was situated directly over the brow. Four three-channel regions were sub-divided to create four regions of interest in the PFC corresponding to the right inferior PFC, right frontopolar PFC, right superior PFC, and right dorsal lateral PFC (Fig. 2A). The remaining 2 × 3 optode patch covered a single region of interest corresponding to the right temporal cortex (Fig. 2B). Consistent placement of the right temporal optodes was accomplished by placing the anterior edge of the 2 × 3 optode patch over each participants interauricular arc and the inferior edge of the patch directly above the T4 10/20 location. In this manner, a significant portion of the frontal-temporal cortical regions involved in social cognition network was simultaneously assessed within both participants.

### Analysis of fNIRS Data

#### Cortical activation analyses

Task related cortical activation was assessed by a general linear model approach^28^. For this analysis, all fNIRS data was pre-processed using Matlab-based functions derived from Homer 2. First, the raw optical density data was motion corrected using a wavelet-based motion artifact removal process^29^. Next, the motion corrected data were band-pass filtered using the low- and high-pass parameters of 0.5 and 0.01 respectively. The filtered optical density data were then converted to oxy-hemoglobin (HbO) and deoxy-hemoglobin (HbR) values by way of the modified Beers-Lambert law^30^,^31^. Only oxy-hemoglobin data were used for the analysis. Next, within each fNIRS recording channel a standardized beta-value was estimated for the cooperation task and inter-block rest period respectively. The task-related activation values were then contrasted with the inter-block rest value, providing an indicator of the degree of change in cortical activation during the task compared to rest. Positive contrast values indicate greater activation during the cooperation task compared to rest. The contrast values for each channel within each region of interest were then averaged together, resulting in five contrast values per participant.

#### Inter-brain coherence analyses

For each region of interest, the cross-correlation between the fNIRS signals generated by each dyad during the cooperation task was measured using wavelet transform coherence. Specifically, channel-wise coherence was calculated using the WTC package (http://noc.ac.uk/using-science/crosswavelet-wavelet-coherence) in Matlab. This method is capable of identifying locally phase-locked behavior between two time-series that might not be discoverable with traditional time series analyses such as Fourier analysis. Of primary interest for our study was the inter-brain coherence that occurred within the task-specific frequency band between periods 3.2 and 12.8 s (corresponding to frequency 0.3 Hz and 0.08 Hz, respectively). Therefore, the mean of all wavelet values within this frequency band was calculated for both cooperation blocks, and the inter-block rest period. Inter-brain coherence values were then averaged amongst each channel that comprised a region of interest. *Task-related coherence* was defined as the mean coherence within blocks one and two of the cooperation task. Next, *coherence increase* was defined as the task-related coherence minus the average coherence during rest 
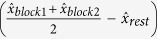
. A positive mean-coherence difference value indicates that the inter-brain coherence between dyads during cooperation was greater than the coherence that occurred during rest. All coherence values were converted to standardized z-values prior to statistical analysis^22^,^23^,^32^.

## Results

### Behavioral Outcomes

**Figure 3:**
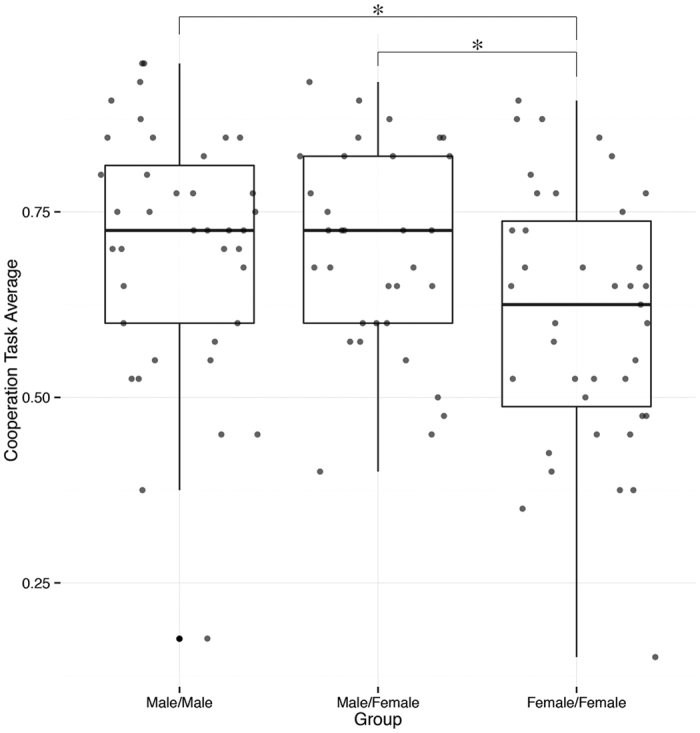
Cooperation performance across dyads. Analysis of variance identified significant performance differences across groups (p = 0.002). Specifically, male/male (p = 0.003) and male/female (p = 0.004) significantly outperformed female/female groups. The bold mid-line within each box and whisker plot provides the median performance for each group. Each box represents the cooperation task performance distribution for 50% of the dyads within each sex pairing. The whiskers extending from each colored box represent the minimum and maximum performance, and the dots represent outlying performance. Outliers were defined as any value less than or equal to 1.5 times the group inter-quartile range.

Behavioral cooperation task performance between male/male, male/female, and female/female dyads was assessed by a three-way analysis of variance. This analysis identified a significant main effect of dyad pairing (*F*(2, 208) = 6.256, *p* = 0.002), indicating that performance differed between groups. Pairwise comparisons indicated that female/female dyads had significantly fewer ‘winning’ trials than both male/male (*t*(151.84) = 3.136, FDR *p* = 0.003) and male/female (*t*(140.19) = 3.117, FDR *p* = 0.004) dyads. These results indicate that for our task, dyads containing at least one male resulted in significantly greater cooperation performance than female only dyads (Fig. 3).

### Cortical Activation Outcomes

**Figure 4:**
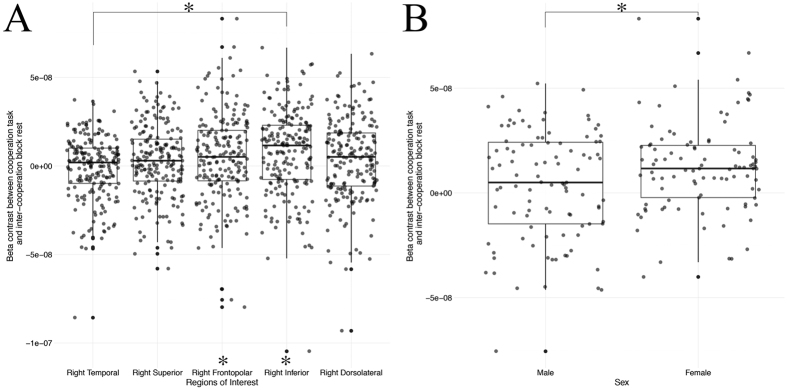
Cortical activation during cooperation. (**A**) Investigation of cortical activation across each region of interest identified significant increases in activation relative to rest within the right frontopolar (p = 0.027) and right inferior (p < 0.001) regions. Pairwise comparisons revealed significant activation differences between the right inferior and right temporal (p < 0.001). (**B**) Female participants elicited significantly greater cortical activation compared to males (p = 0.013).

First, we identified those cortical regions of interest that responded significantly to our task in individual participants. To achieve this objective, we conducted FDR corrected one-sample t-tests on GLM *task vs. rest* beta contrast values for all participants within each of our five regions of interest. This analysis revealed significant activation in the right frontopolar (*t*(195) = 2.569, FDR *p* = 0.027) and right inferior (*t*(194) = 4.497, FDR *p* < 0.001) prefrontal cortices (Fig. 4A). Pairwise comparisons of activation across each region highlighted a significant difference between the right inferior and right temporal regions (*t*(357) = 4.113, FDR *p* < 0.001). Next, we further investigated activation differences across males and females within the right frontopolar and right inferior regions of interest. The contrast values from these regions were submitted to a 2 (sex) × 3 (group: male/male, male/female, female/female) × 2 (regions of interest) ANOVA. This analysis revealed a significant main effect of sex (*F*(1, 188) = 6.189, *p* = 0.013), indicating that, for our task, female participants exhibited significantly greater cortical activation than males within the right frontopolar and inferior prefrontal cortices (Fig. 4B). No other comparisons were significant.

In order to investigate the degree to which cortical activations differed across males and females separately, we conducted identical analyses as reported above on each group individually. FDR corrected one-sample t-tests indicated that females exhibited significant activation within the right frontopolar (*t*(94) = 2.915, FDR *p* = 0.014), right inferior prefrontal (*t*(93) = 5.138, *p* < 0.001), and the right dorsolateral prefrontal (*t*(94) = 3.452, *p* = 0.004) cortices. A 3 (regions of interest) × 2 (group: male/female, female/female) ANOVA did not reveal any significant differences in cortical response across regions of interest or group. Moreover, no significant effects were identified for males alone.

### Inter-brain Coherence Outcomes

**Figure 5:**
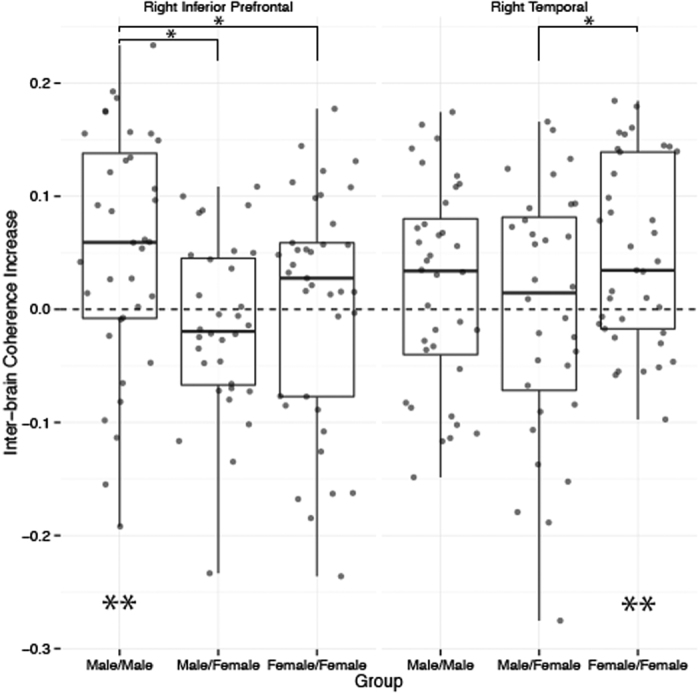
Inter-brain coherence increase within the right inferior and right temporal cortices. A significant increase in inter-brain coherence was identified in male/male groups’ right inferior pre-frontal cortex (p = 0.020), and was significantly greater than male/female (p = 0.003) and female/female (p = 0.046) groups. Moreover, a significant increase in inter-brain coherence was identified in female/female groups right temporal cortex (p = 0.005). Female/female coherence in this region was significantly greater than male/female group coherence (p = 0.039).

As reported previously^22^,^23^,^24^,^33^,^34^,^35^,^36^,^37^,^38^,^39^,^40^, analysis of inter-brain coherence increase effectively identifies regions of the cortex that become significantly correlated between members of a dyad during cooperation. In order to replicate previous results and identify cortical regions of interest for further investigation as reported below, we first analyzed the patterns of coherence increase that arose across each dyad type (male/male, male/female, female/female). To that end, FDR corrected one-sample t-tests were conducted on the coherence increase values for each group across each region of interest. This analysis identified significant coherence increase in male/male pairs within the right inferior prefrontal cortex (*t*(35) = 2.197, FDR *p* = 0.020). Moreover, female/female dyads exhibited significant coherence increase within the right temporal cortex (*t*(36) = 3.859, FDR *p* = 0.005). Based on these outcomes, our regions of interest for all remaining inter-brain coherence analyses were reduced to the right inferior prefrontal and right temporal cortices. Next, the coherence increase values were submitted to a 3 (group) × 2 (regions of interest) ANOVA. This analysis revealed a significant main effect of group (*F*(2, 204) = 4.033, *p* = 0.019), driven by significantly less coherence increase in male/female compared to male/male (*t*(133.18) = 2.672, FDR *p* = 0.023) and female/female (*t*(132.58) = 2.162, FDR *p* = 0.050) groups. Furthermore, this analysis revealed a significant group × region interaction (*F*(2, 204) = 3.021, *p* = 0.050). Independent-sample t-tests applied between all groups within both regions of interest highlighted significantly greater coherence increase in male/male dyads right inferior prefrontal cortex compared to both female/female (*t*(70.891) = 2.025, FDR *p* = 0.046) and male/female (*t*(63.631) = 3.12, FDR *p* = 0.003) dyads. Conversely, the coherence increase in female/female dyads right temporal region was significantly greater than male/female dyads (*t*(56.141) = 2.096, FDR *p* = 0.039), but did not differ significantly from male/male dyads (t(69.764) = 1.407, FDR *p* > 0.05) (Fig. 5).

### Relationship Between Cooperation Behavior and fNIRS Data

#### Cortical activation

In order to identify underling mechanistic processes that may connect fNIRS signals with real-time cooperation behavior, we investigated the relationship between task performance and cortical activation. Linear regression was used to assess the degree to which participant’s cooperation task performance predicted cortical activation. No significant relationship between cortical activation and cooperation performance was identified.

#### Task-related inter-brain coherence

**Figure 6:**
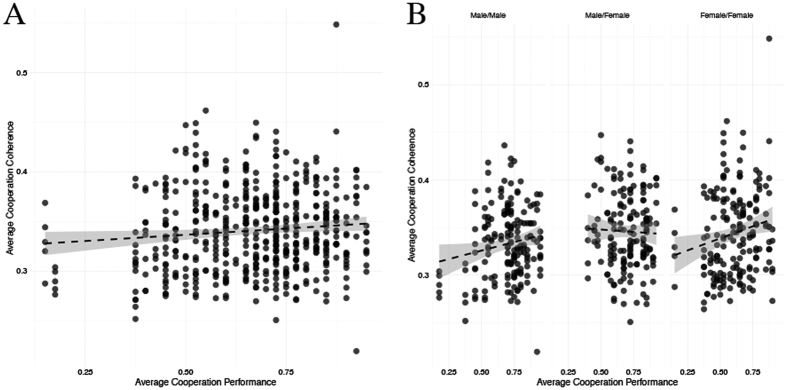
Relationship between cooperation performance and task-related inter-brain coherence. (**A**) Cooperation performance significantly predicts inter-brain coherence (*r* = 0.603*, p* = 0.024) across all regions. (**B**) The relationship between cooperation performance and inter-brain coherence was significant for male/male (*r* = 0.862, *p* = 0.035) and female/female (*r* = 1.195, *p* = 0.012) groups. This relationship was positive within these groups, indicating that greater behavioral performance coincided with enhanced inter-brain coherence. Conversely, this relationship within male/female pairs was non-significant (*p* = 0.537, *r* = −0.147).

We employed an identical linear regression analysis as reported above to assess the relationship between cooperation performance and inter-brain coherence. Of primary interest was the strength of this relationship that occurred during cooperation. Thus, for this analysis we used cooperation task performance to predict task-related inter-brain coherence within each region of interest (i.e., mean coherence within each region during blocks 1 and 2 of the cooperation task). This analysis identified a positive relationship between performance and task-related coherence, indicating that greater task performance coincided with greater task-related inter-brain coherence (*r* = 0.603, *p* = 0.024) (Fig. 6A). Next, we conducted a series of identical linear regression analyses on each dyad type (male/male, male/female, female/female) individually. These analyses identified significant positive relationships between cooperation performance and task-related coherence across all regions of interest within male/male (*r* = 0.862, *p* = 0.035) and female/female dyads (*r* = 1.195, *p* = 0.012). This relationship was not significant for male/female dyads (Fig. 6B). When further stratified across the regions of interest, a significant relationship between cooperation task performance and inter-brain coherence was identified within the right temporal region for female/female dyads (*r* = 0.323, *p* = 0.028). No other comparisons were significant.

## Discussion

Here, we employed fNIRS hyperscanning to image multiple regions of the frontal-temporal social network of same- and mixed-sex dyads during an interactive cooperation game. Our results indicate that sex, as a biological variable, significantly influences cooperation behavior, cortical activations, location of inter-brain coherence, and the interactions between inter-brain coherence and cooperation behavior. These results provide new and fundamental information regarding the underlying cognitive sources of cooperation differences between males and females.

Interestingly, groups containing at least one male performed significantly better than female/female dyads on our cooperation task. These results indicate that males engaged a behavioral strategy that benefited cooperation performance, and that only one member of a dyad needed to engage this strategy for the dyad as a whole to be relatively more successful during cooperation. In terms of task-related cortical activation, our data indicate that the cooperation task elicited significant prefrontal activity, although follow-up analyses indicated that this effect was driven by activation in female participants. The cortical regions demonstrating such cooperation task-related activation coincide with Brodmann Area 10 (BA10). Based on our current understanding of the neural correlates of social cognition, significant activation in this region may be related to multitasking (i.e., retrieval of higher order goals) and theory of mind^41^,^42^. More specifically, BA10 has been shown to activate during passive viewing of cooperative interactions^43^, to be engaged during tasks involving cooperation in two-person decision-making games^44^, and to subserve person perception and mentalization^1^. In conjunction with behavioral cooperation performance reported above, it is noteworthy that task-related activation in BA10 was greater in females, and coincided with female’s poorer behavioral performance. Thus, we may speculate that females required increased executive functioning processes throughout the cooperation task, which may have negatively influenced their cooperation performance in terms of person perception and mentalization. However, by nature of a single-person cortical activation analysis, the degree to which two members of an interacting dyad simultaneously engage the same cognitive processes remains unknown. Contrary to the above individual task-related results, significant inter-brain coherence in BA10 during cooperation was only identified within male/male dyads. These results are important, as they provide evidence that both members of male/male dyads similarly engaged this region throughout our cooperation task. Thus, we hypothesize that male/male dyads engaged a cognitive strategy wherein both players attempted to understand their partner’s intentions and motives, which was sustained by BA10. The coincident engagement of this region between both members of a male/male dyad may have helped drive superior behavioral performance throughout our cooperation task and resulted in stronger inter-brain coherence within this region.

Besides revealing significant behavioral as well as task-related and inter-brain coherence patterns in prefrontal cortex, our data also show significant effects in regions of the temporal cortex overlapping with BA21 & 22. These areas have been implicated in social perception, action observation, and theory of mind^45^. For instance, Yang and colleagues^23^ highlight the right superior temporal sulcus as an important component in “temporal predictive encoding of human behavior” and “temporal integration of the key elements in the environment”. Furthermore, the posterior superior temporal cortex has been described as the “region considered most central to the recognition of human actions” in terms of biological motion, action processing, and the representation of specific actions^20^. For our cooperation task, we observed significantly increased inter-brain coherence in temporal cortex for female/female dyads. Such pattern may suggest that female players relied predominantly on action-centered social cognitive processes during cooperation. While adherence to this cognitive strategy between females positively influenced cooperation performance, overall female/female cooperation performance was significantly lower than male/male and male/female performance. Thus, these data suggest that action-centered cooperation strategies may be less optimal for our cooperation task.

Male/female dyads performed significantly better on our cooperation task compared to female/female dyads, and comparable to male/male dyads. Unlike male/male dyads, male/female cooperation performance was not accompanied by significant task-related increase in inter-brain coherence in any cortical region that we targeted. Furthermore, unlike same-sex dyads, there was no significant association between task-related inter-brain coherence and cooperation task performance in mixed-sex pairs. We hypothesize that the lack of significant inter-brain coherence in mixed-sex dyads may be indicative of different cognitive strategies employed by males and females during cooperation. Notably, the results of Cheng and colleagues^23^, which employed the same cooperation task used here, identified significant coherence within mixed- but not same-sex dyads. However, because Cheng and colleagues focused solely on the prefrontal cortex, the role of sex on inter-brain coherence patterns within their participant dyads’ right temporal cortex is unknown. The discrepancy between their results and ours may simply be due to sample size differences between studies; Cheng and colleagues^23^ reported findings from 14 male/male, 15 female/female, and 16 male/female dyads, which represents less than half of our sample size within each group (i.e., 39 male/male, 38 female/female, 34 male/female). Alternatively, an intriguing possibility is that cultural differences between study populations drawn from predominantly Asian compared to Western societies led to differential patterns of inter-brain coherence during cooperation^46^. Future research is needed to further elucidate the role of culture and society on neural and behavioral signatures of cooperation. Nevertheless, our results suggest that the lack of significant inter-brain coherence within frontal-temporal brain regions does not adversely affect behavioral cooperation performance. We may speculate that high behavioral cooperation performance may be maintained when only one member of an interacting dyad engages an optimal cooperation strategy. Future research is needed to determine the extent to which social behavior may be influenced when members of an interacting dyad engage different cognitive strategies.

Our data highlight predictive relationships between inter-brain coherence and cooperation performance within same- but not mixed-sex dyads. Importantly, this relationship was significant for all dyad types combined, as well as for both same-sex dyads individually. Furthermore, when stratified across dyad types and regions of interest, this relationship within same-sex dyads closely mirrors the patterns of inter-brain coherence increases reported above. That is, within female/female dyads, the relationship between inter-brain coherence and behavioral cooperation performance was significant within the right temporal brain region only. Within male/male dyads, the relationship between inter-brain coherence and cooperation performance was strongest in the right inferior prefrontal region. However, unlike for female/female dyads, this analysis narrowly missed the statistical rejection criterion (i.e., *p* = 0.062). These findings suggest that greater adherence to sex-specific cognitive strategies throughout our cooperation task, which manifest through greater inter-brain coherence in different regions of the frontal-temporal network, coincided with better behavioral performance relative to dyads that employed different strategies and thus showed less task-related inter-brain coherence.

Finally, our findings demonstrate that the location of cortical activation and inter-brain coherence do not necessarily coincide. As overlapping increases in both measures was identified in the right inferior prefrontal cortex, significant cortical activation with no inter-brain coherence was identified in the frontopolar region. Moreover, significant increases in female/female inter-brain coherence without coincident cortical activation was identified in the right temporal cortex. These results highlight the utility of assessing multiple aspects of fNIRS hyperscanning data when investigating social cognition. For example, analysis of cortical activations alone would fail to identify the role of the right temporal region throughout cooperation, and would focus results on sex differences in executive functioning during cooperation. Conversely, sole focus on inter-brain coherence would fail to identify sex differences in cortical activation.

## Limitations and Future Directions

The results of this study provide new, fundamental information regarding the influence of sex on concurrent neural and behavioral signatures of cooperation within multiple regions of the social cognition network. While our study is the first to detail the signatures of cooperation-related inter-brain coherence that emerges within multiple regions of the frontal and temporal cortices, there is little doubt that important aspects of social cognition are associated with other brain regions that, due to the physical limitations of our fNIRS device, we were unable to target. Emergent advances in fNIRS technology that provide more recording channels should make it possible to cover more regions of the brain in future hyperscanning studies focused on social cognition. Future hyperscanning research that investigates signatures of inter-brain coherence occurring in the context of ecologically valid cooperation tasks will also be valuable in advancing our knowledge in this area. The cooperation task that we used was originally developed by our group^22^ as a basic computer-based form of cooperation, and it has been used elsewhere for the same purpose^23^. However, it is possible that naturalistic cooperation may elicit different signatures of cortical activation and inter-brain coherence. Fortunately, the portability of newer fNIRS devices makes it possible to interrogate these processes in the context of more real-world cooperation tasks. Finally, it is important that future efforts be made to optimize approaches for quantifying the relationship between inter-brain coherence and cooperation behavior. Here, we simply regressed the average task-related coherence that occurred in both task blocks onto cooperation task performance. We see this as an advantage, as this approach may easily be adapted to real-time feedback applications. That is, the relationship between a pairs’ average coherence and task performance may be calculated and displayed in real-time. However, this method differs from Cheng and colleagues^23^, in which a correlation was made between coherence increase (i.e., task - rest) and coherence difference (i.e., block 2 - block 1). Future research is needed to evaluate, compare and optimize these and other approaches.

## Additional Information

**How to cite this article**: Baker, J. M. *et al*. Sex differences in neural and behavioral signatures of cooperation revealed by fNIRS hyperscanning. *Sci. Rep.* **6**, 26492; doi: 10.1038/srep26492 (2016).

## References

[b1] Amodio D. M. & Frith C. D. Meeting of minds: the medial frontal cortex and social cognition. Nature Reviews Neuroscience 7, 268–277 (2006). 16552413 10.1038/nrn1884

[b2] Saxe R. Uniquely human social cognition. Curr Opin Neurobiol 16, 235–239 (2006). 16546372 10.1016/j.conb.2006.03.001

[b3] Behrens T. E., Hunt L. T. & Rushworth M. F. The computation of social behavior. Science 324, 1160–1164 (2009). 19478175 10.1126/science.1169694

[b4] Yun K., Watanabe K. & Shimojo S. Interpersonal body and neural synchronization as a marker of implicit social interaction. Scientific reports 2, 1–8, doi: 10.1038/srep00959 (2012). PMC3518815 23233878

[b5] Johnson S. C. . Neural correlates of self‐reflection. Brain 125, 1808–1814 (2002). 12135971 10.1093/brain/awf181

[b6] Zysset S., Huber O., Ferstl E. & von Cramon D. Y. The anterior frontomedian cortex and evaluative judgment: an fMRI study. NeuroImage 15, 983–991 (2002). 11906238 10.1006/nimg.2001.1008

[b7] Schmitz T. W., Kawahara-Baccus T. N. & Johnson S. C. Metacognitive evaluation, self-relevance, and the right prefrontal cortex. NeuroImage 22, 941–947 (2004). 15193625 10.1016/j.neuroimage.2004.02.018

[b8] Walton M. E., Devlin J. T. & Rushworth M. F. Interactions between decision making and performance monitoring within prefrontal cortex. Nat Neurosci 7, 1259–1265 (2004). 15494729 10.1038/nn1339

[b9] Brown J. W. & Braver T. S. Learned predictions of error likelihood in the anterior cingulate cortex. Science 307, 1118–1121 (2005). 15718473 10.1126/science.1105783

[b10] Coricelli G. . Regret and its avoidance: a neuroimaging study of choice behavior. Nat Neurosci 8, 1255–1262 (2005). 16116457 10.1038/nn1514

[b11] Camille N. . The involvement of the orbitofrontal cortex in the experience of regret. Science 304, 1167–1170 (2004). 15155951 10.1126/science.1094550

[b12] Fellows L. K. & Farah M. J. Different underlying impairments in decision-making following ventromedial and dorsolateral frontal lobe damage in humans. Cerebral cortex 15, 58–63 (2005). 15217900 10.1093/cercor/bhh108

[b13] Saygin A. P. Superior temporal and premotor brain areas necessary for biological motion perception. Brain 130, 2452–2461 (2007). 17660183 10.1093/brain/awm162

[b14] Pelphrey K. A., Morris J. P. & Mccarthy G. Grasping the intentions of others: the perceived intentionality of an action influences activity in the superior temporal sulcus during social perception. Journal of cognitive neuroscience 16, 1706–1716 (2004). 15701223 10.1162/0898929042947900

[b15] Decety J. & Lamm C. The role of the right temporoparietal junction in social interaction: how low-level computational processes contribute to meta-cognition. The Neuroscientist 13, 580–593, doi: 10.1177/1073858407304654 (2007). 17911216

[b16] Van Overwalle F. Social cognition and the brain: a meta‐analysis. Human brain mapping 30, 829–858 (2009). 18381770 10.1002/hbm.20547 PMC6870808

[b17] Saxe R. & Kanwisher N. People thinking about thinking people: the role of the temporo-parietal junction in “theory of mind”. NeuroImage 19, 1835–1842 (2003). 12948738 10.1016/s1053-8119(03)00230-1

[b18] Ramnani N. & Miall R. C. A system in the human brain for predicting the actions of others. Nat Neurosci 7, 85–90 (2004). 14699420 10.1038/nn1168

[b19] Grosbras M.-H. & Paus T. Brain networks involved in viewing angry hands or faces. Cerebral Cortex 16, 1087–1096 (2006). 16221928 10.1093/cercor/bhj050

[b20] Thompson J. & Parasuraman R. Attention, biological motion, and action recognition. NeuroImage 59, 4–13 (2012). 21640836 10.1016/j.neuroimage.2011.05.044

[b21] Schilbach L. . Toward a second-person neuroscience. Behavioral and Brain Sciences 36, 393–414 (2013). 23883742 10.1017/S0140525X12000660

[b22] Cui X., Bryant D. M. & Reiss A. L. NIRS-based hyperscanning reveals increased interpersonal coherence in superior frontal cortex during cooperation. NeuroImage 59, 2430–2437, doi: 10.1016/j.neuroimage.2011.09.003 (2012). 21933717 PMC3254802

[b23] Cheng X., Li X. & Hu Y. Synchronous brain activity during cooperative exchange depends on gender of partner: A fNIRS‐based hyperscanning study. Human brain mapping 36, 2039–2048 (2015). 25691124 10.1002/hbm.22754 PMC6869051

[b24] Babiloni F. & Astolfi L. Social neuroscience and hyperscanning techniques: past, present and future. Neuroscience & Biobehavioral Reviews 44, 76–93, doi: 10.1016/j.neubiorev.2012.07.006 (2012). 22917915 PMC3522775

[b25] Bedell J. & Sistrunk F. Power, opportunity costs, and sex in a mixed-motive game. Journal of personality and social psychology 25, 219–226, doi: 10.1037/h0033947 (1973).

[b26] Van Vugt M., De Cremer D. & Janssen D. P. Gender differences in cooperation and competition the Male-Warrior hypothesis. Psychological Science 18, 19–23 (2007). 17362372 10.1111/j.1467-9280.2007.01842.x

[b27] Balliet D., Li N. P., Macfarlan S. J. & Van Vugt M. Sex differences in cooperation: a meta-analytic review of social dilemmas. Psychological bulletin 137, 881 (2011). 21910518 10.1037/a0025354

[b28] Plichta M., Heinzel S., Ehlis A.-C., Pauli P. & Fallgatter A. Model-based analysis of rapid event-related functional near-infrared spectroscopy (NIRS) data: a parametric validation study. NeuroImage 35, 625–634 (2007). 17258472 10.1016/j.neuroimage.2006.11.028

[b29] Molavi B. & Dumont G. A. Wavelet-based motion artifact removal for functional near-infrared spectroscopy. Physiological measurement 33, 259 (2012). 22273765 10.1088/0967-3334/33/2/259

[b30] Strangman G., Franceschini M. A. & Boas D. A. Factors affecting the accuracy of near-infrared spectroscopy concentration calculations for focal changes in oxygenation parameters. NeuroImage 18, 865–879 (2003). 12725763 10.1016/s1053-8119(03)00021-1

[b31] Jobsis F. F. Noninvasive, infrared monitoring of cerebral and myocardial oxygen sufficiency and circulatory parameters. Science 198, 1264–1267 (1977). 929199 10.1126/science.929199

[b32] Chang C. & Glover G. H. Time–frequency dynamics of resting-state brain connectivity measured with fMRI. NeuroImage 50, 81–98 (2010). 20006716 10.1016/j.neuroimage.2009.12.011 PMC2827259

[b33] Anders S., Heinzle J., Weiskopf N., Ethofer T. & Haynes J.-D. Flow of affective information between communicating brains. NeuroImage 54, 439–446 (2011). 20624471 10.1016/j.neuroimage.2010.07.004 PMC3081064

[b34] Babiloni F. . In *Engineering in Medicine and Biology Society, 2007. EMBS 2007. 29th Annual International Conference of the IEEE*. 4957-4960 (IEEE). 10.1109/IEMBS.2007.4353453 18003119

[b35] Dumas G., Lachat F., Martinerie J., Nadel J. & George N. From social behaviour to brain synchronization: review and perspectives in hyperscanning. IRBM 32, 48–53 (2011).

[b36] Dumas G., Nadel J., Soussignan R., Martinerie J. & Garnero L. Inter-brain synchronization during social interaction. Plos one 5, e12166 (2010). 20808907 10.1371/journal.pone.0012166 PMC2923151

[b37] Hasson U., Nir Y., Levy I., Fuhrmann G. & Malach R. Intersubject synchronization of cortical activity during natural vision. Science 303, 1634–1640 (2004). 15016991 10.1126/science.1089506

[b38] Bilek E. . Information flow between interacting human brains: Identification, validation, and relationship to social expertise. Proceedings of the National Academy of Sciences 112, 5207–5212 (2015). 10.1073/pnas.1421831112 PMC4413334 25848050

[b39] Lindenberger U., Li S.-C., Gruber W. & Müller V. Brains swinging in concert: cortical phase synchronization while playing guitar. BMC neuroscience 10, 22 (2009). 19292892 10.1186/1471-2202-10-22 PMC2662862

[b40] Montague P. R. . Hyperscanning: simultaneous fMRI during linked social interactions. NeuroImage 16, 1159–1164 (2002). 12202103 10.1006/nimg.2002.1150

[b41] Roca M. . The role of Area 10 (BA10) in human multitasking and in social cognition: a lesion study. Neuropsychologia 49, 3525–3531 (2011). 21930138 10.1016/j.neuropsychologia.2011.09.003

[b42] Chahine G., Diekhof E. K., Tinnermann A. & Gruber O. On the role of the anterior prefrontal cortex in cognitive ‘branching’: An fMRI study. Neuropsychologia, Nsyd1500071, doi: 10.1016/j.neuropsychologia.2015.08.018 (2015). 26300386

[b43] Proverbio A. M. . Neural coding of cooperative vs. affective human interactions: 150 ms to code the action’s purpose. Plos one 6, e22026, doi: 10.1371/journal.pone.0022026 (2011). 21760948 PMC3131384

[b44] McCabe K., Houser D., Ryan L., Smith V. & Trouard T. A functional imaging study of cooperation in two-person reciprocal exchange. Proceedings of the National Academy of Sciences 98, 11832–11835 (2001). 10.1073/pnas.211415698 PMC58817 11562505

[b45] Yang D. Y.-J., Rosenblau G., Keifer C. & Pelphrey K. A. An integrative neural model of social perception, action observation, and theory of mind. Neuroscience & Biobehavioral Reviews 51, 263–275 (2015). 25660957 10.1016/j.neubiorev.2015.01.020 PMC4940188

[b46] Han S. & Ma Y. Cultural differences in human brain activity: A quantitative meta-analysis. NeuroImage 99, 293–300 (2014). 24882220 10.1016/j.neuroimage.2014.05.062

