# Corrigendum: Sex differences in neural and behavioral signatures of cooperation revealed by fNIRS hyperscanning

**DOI:** 10.1038/srep30512

**Published:** 2016-08-19

**Authors:** Joseph M. Baker, Ning Liu, Xu Cui, Pascal Vrticka, Manish Saggar, S. M. Hadi Hosseini, Allan L. Reiss

Scientific Reports 6: Article number: 2649210.1038/srep26492; published online: 06 08 2016; updated: 08 19 2016.

In this Article, the authors mistakenly used the abbreviation “*r*” instead of “*b*” to refer to the beta coefficients resulting from the analysis of the relationship between cooperation behavior and task-related inter-brain coherence. All statistical outcomes and their interpretations remain unaffected and unchanged. As a result,

“This analysis identified a positive relationship between performance and task-related coherence, indicating that greater task performance coincided with greater task-related inter-brain coherence (*r* = 0.603, *p* = 0.024) (Fig. 6A). Next, we conducted a series of identical linear regression analyses on each dyad type (male/male, male/female, female/female) individually. These analyses identified significant positive relationships between cooperation performance and task-related coherence across all regions of interest within male/male (*r* = 0.862, *p* = 0.035) and female/female dyads (*r* = 1.195, *p* = 0.012). This relationship was not significant for male/female dyads (Fig. 6B). When further stratified across the regions of interest, a significant relationship between cooperation task performance and inter-brain coherence was identified within the right temporal region for female/female dyads (*r* = 0.323, *p* = 0.028). No other comparisons were significant.”

should read:

“This analysis identified a positive relationship between performance and task-related coherence, indicating that greater task performance coincided with greater task-related inter-brain coherence (*b* = 0.603, *p* = 0.024) (Fig. 6A). Next, we conducted a series of identical linear regression analyses on each dyad type (male/male, male/female, female/female) individually. These analyses identified significant positive relationships between cooperation performance and task-related coherence across all regions of interest within male/male (*b* = 0.862, *p* = 0.035) and female/female dyads (*b* = 1.195, *p* = 0.012). This relationship was not significant for male/female dyads (Fig. 6B). When further stratified across the regions of interest, a significant relationship between cooperation task performance and inter-brain coherence was identified within the right temporal region for female/female dyads (*b* = 0.323, *p* = 0.028). No other comparisons were significant.”

In the legend of Figure 6,

“(**A**) Cooperation performance significantly predicts inter-brain coherence (*r* = 0.603*, p* = 0.024) across all regions. (**B**) The relationship between cooperation performance and inter-brain coherence was significant for male/male (*r* = 0.862, *p* = 0.035) and female/female (*r* = 1.195, *p* = 0.012) groups. This relationship was positive within these groups, indicating that greater behavioral performance coincided with enhanced inter-brain coherence. Conversely, this relationship within male/female pairs was non-significant (*p* = 0.537, *r* = −0.147).”

should read:

“(**A**) Cooperation performance significantly predicts inter-brain coherence (*b* = 0.603*, p* = 0.024) across all regions. (**B**) The relationship between cooperation performance and inter-brain coherence was significant for male/male (*b* = 0.862, *p* = 0.035) and female/female (*b* = 1.195, *p* = 0.012) groups. This relationship was positive within these groups, indicating that greater behavioral performance coincided with enhanced inter-brain coherence. Conversely, this relationship within male/female pairs was non-significant (*b* = 0.537, *r* = −0.147).”

